# Primary chondrosarcoma of the breast: a case presentation and review of the literature

**DOI:** 10.1186/1477-7819-11-208

**Published:** 2013-08-21

**Authors:** Sanaa Errarhay, Mohamed Fetohi, Samia Mahmoud, Hanane Saadi, Chahrazed Bouchikhi, Abdelaziz Banani

**Affiliations:** 1 Department of Obstetrics and Gynecology, University Hospital Hassan II, Sidi Harazem Road, Fez 30000, Morocco; 2 Department of Oncology, Moulay Ismail Hospital, Ville Nouvelle, Meknes 50020, Morocco

**Keywords:** Chondrosarcoma, Breast, Sarcoma

## Abstract

Mammary sarcomas are uncommon tumors. When tumors like malignant cystosarcomaphyllodes and metaplastic carcinoma, where malignant cartilaginous areas may be present, are excluded, only nine cases have been reported to date.We report another case of primary chondrosarcoma of the breast here. A 24-year-old Mediterranean woman presented with a painful mass in the right breast and a physical examination revealed a palpable mass. An incisional biopsy was performed and primary chondrosarcoma was diagnosed based on histological examination. Our patient underwent a mastectomy. A preoperative clinical and cytological diagnosis of chondrosarcoma, even though possible in a few cases, is usually not attained due to its similarclinical behavior with other breast tumors.

## Background

Pure sarcomas are very uncommon tumors of the breast, representing about 0.5% of all mammary tumors. These are rare tumors that must be considered in the differential diagnosis of breast tumors when chondrosarcomatous areas are involved. It is important that they are recognized as a separate entity from the more common breast carcinomas and the difference in behavior of these two tumors is taken into accountwhen planning therapy.

## Case presentation

**Figure 1:**
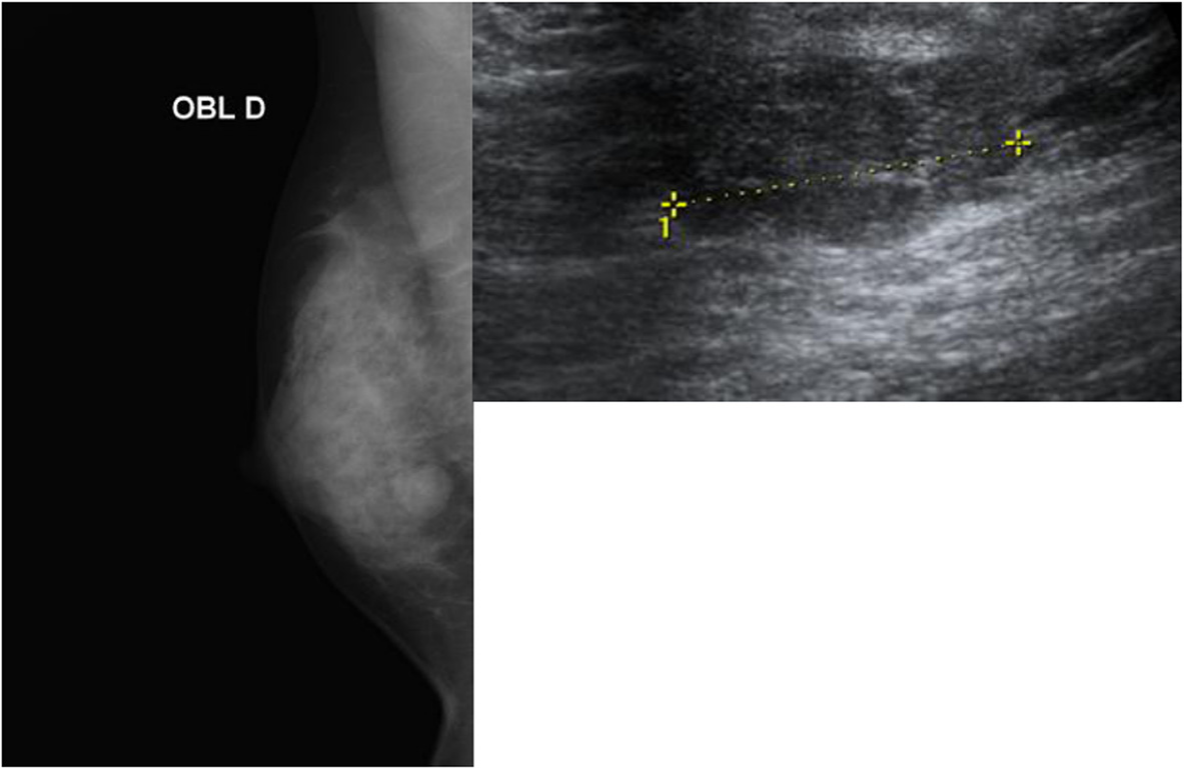
**Mammography showing a round,well delineated opaque image in the lower outer quadrant of the right breast.** Ultrasound showing a well delineated bilobed tissue lesion measuring 15 mm.

**Figure 2:**
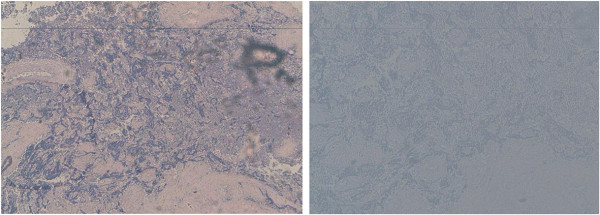
Round or oval tumor cell proliferation containing various differentiated cartilaginous tissues.

A 24-year-old Mediterranean woman presented with a painful mass in the right breast that had increased in size over a period of five months. The woman had no medical history, and no family history, of breast cancer. A physical examination revealed a palpable mass in her right breast measuring 10 mm to 15 mm in size, not fixed to underlying tissues and not involving the overlying skin. Her contralateral breast and axilla were normal on clinical examination. On mammography, the mass was well demarcated (Figure 1). Her liver ultrasound scan, chest X-ray and bone scintigraphy were normal. An incisional biopsy was performed and the histological examination showed abundant cartilaginous proliferation varying from mature cartilage to poorly differentiated areas (Figure 2). An immunohistochemical study was performed and immunoreactivity was detected for vimentin but not for AE1/AE3, CK7, and estrogen and progesterone receptors. There was also no overexpression of the HER-2/neuoncoprotein. A final diagnosis of chondrosarcoma of the breast was made with the absence of phyllodes tumor or carcinoma in the whole tumor. Our patient underwent a mastectomy.

## Discussion

Primary breast sarcomas are a highly heterogenous group of tumors. A majority of these tumors are malignant fibrous histiocytoma, fibrosarcoma, liposarcoma and less commonly angiosarcoma, rhabdomyosarcoma, dermatofibrosarcoma, desmoids tumors and so on. A primary chondrosarcoma of the breast is an extremely rare entity. It contains chondrosarcomatoid sectors which arisefrom mammary tissue. Only nine cases of pure chondrosarcoma have been reported: Kennedy and Biggart reported the first case in 1967, Beltaos and Banerjee reported two cases in 1978, and Thilagavathi brought the fourth case in 1992 [1-3]. The fifth case was reported by Guymar *et al*. in 2001 and the 10th case is reported here [4]. The prognosis of chondrosarcomatous breast tumors is not fully known, because many of the reported cases are difficult to analyze owing to the lack of detailed clinical or morphological information. These tumors are usually large-sized and occur in women over 40 years old. These tumors usually do not invade the overlying skin [1]. Axillary adenopathy are found in 14 to 29% of the cases, most of which are reactive hyperplasia. The present case substantiates the clinical findings of previously reported cases [1]. To diagnose a primary chondrosarcoma of the breast, a non-mammary primary site has to be excluded clinically and histologically. Differentiation from metaplastic carcinoma is possible by the absence of direct transition between the carcinomatous and the mesenchymal component in the formermetaplastic carcinoma. Further, although the sarcoma-like elements in metaplastic carcinoma acquire vimentin positivity, they still retain epithelial markers [5].

Differentiation from malignant cystosarcomaphyllodes with predominant chondrosarcomatoid components can be extremely difficult. Most mammary tumors with areas of chondroid metaplasia havea benign histological appearance. Cystosarcomaphyllodes displaying a chondrosarcomatous element are very rare.

The majority of information that guides treatment management consists of retrospective chart reviews, anecdotal experience, and case reports. Surgery remains the choice of treatment for most sarcomatoid tumors [6]. Multimodality treatment may decrease local and systemic recurrence rates of somatic sarcomas, but results are inconclusive in patients with breast sarcomas [7,8]. The role of chemotherapy and radiotherapy is not yet established because of the limited number of cases reported so far. The tumor was negative for any of the hormonal receptors. This supports the theory that adjuvant therapy with estrogen antagonists and other hormone manipulations have no role in the treatment of mammary sarcomas.

The adjuvant treatment can decrease the rates of local and systematic recurrences, but the results are not significant because of the rarity of this pathological entity and the small number of cases reported, which makes the evaluation of the role of chemotherapy and radiotherapy in primary breast chondrosarcoma more difficult [7,9,10]. In Emad *et al*.’s study of malignant matrix-producing breast tumors (MP-MBC), they concluded that most MP-MBCs are variants of metaplastic breast carcinoma (MBC) with predominant mesenchymal components behaving similarly to ductal carcinomas. Nevertheless, these tumors cannot be managed differently from other forms of triple-negative breast cancer as there exist limited data on their response to systemic therapy [11].

## Conclusions

Primary sarcomas of the breast are rare tumors and it is important that these tumors are recognized as a separate entity from the more common breast carcinomasand the difference in behavior of these two tumors iskept in mindwhen planning therapy.

## Consent

Written informed consent was obtained from the patient for publication of this manuscript and any accompanying images. A copy of the written consent is available for review by the Editor-in-Chief of this journal.

## Competing interests

The authors declare that they have no competing interests.

## Authors’ contributions

ES designed and wrote the paper. FMcarried outthe medical treatment. SM and SH performed thesurgery. BC provided the pathological diagnosis. BA designed the paper. All authors read and approved the final manuscript.

## References

[B1] Kennedy T Biggart JD Sarcoma of the breast Br J Cancer 1967 11 635 644 10.1038/bjc.1967.74 4294611 PMC2008190

[B2] Beltaos E Banerjee TK Chondrosarcoma of the breast: a case report of two cases Am J ClinPathol 1979 11 345 349 10.1093/ajcp/71.3.345 433839

[B3] Thilagavath G Subramanian S Samuel AV Rani U Somasundaram C Primary chondrosarcoma of the breast J Indian Med Assoc 1992 11 16 17 1593140

[B4] Guymar S Ferlicot S Genestie C Genestie C Gelberg JJ Blondon J Le Charpentier Y Breast chondrosarcoma: a case report and review Ann Pathol 2001 11 168 171 11373590

[B5] Vandenhaute B Validire P Veillex C Voelh P Zafrani B Breast carcinoma with chondroid metaplasia Ann Pathol 1995 11 53 58 7702669

[B6] Callery CD Rosen P Kinne DW Sarcome du sein. Une étude de 32 patients présentant la réestimation de la classification et de la thérapie Ann Surg 1985 11 527 532 10.1097/00000658-198504000-00020 3977455 PMC1250744

[B7] Rosen PP Rosen PP Sarcoma Rosen’s Breast Pathology 2001 Philadelphia: Lippincott Williams and Wilkins 813

[B8] Van Deurzen CH Lee AH Gill MS Menke-Pluijmers MB Jager A Ellis IO Metaplastic breast carcinoma:tumourhistogenesis or dedifferentiation? J Pathol 2011 11 434 437 10.1002/path.2872 21462188

[B9] Tseng WH Martinez SR Metaplastic breast cancer: to radiate or not to radiate? Ann SurgOncol 2011 11 94 103 10.1245/s10434-010-1198-6 PMC3018259 20585866

[B10] Gurleyik E Yildirim U Gunal O Pehlivan M Malignant mesenchymal tumor of the breast: primary chondrosarcoma Breast Care 2009 11 101 103 10.1159/000212101 20847886 PMC2931068

[B11] Rakha E Tan PH Shaaban A Tse GM Esteller FC Van Deurzen CH Purnell D Stotter A Chan T Yamaguchi R Dodwell D Jager A Soler MT Juneinah E Plaza ML Hodi Z McCulloch T Lee AH Ellis IO Do primary mammary osteosarcoma and chondrosarcoma exist? a review of a large multi-institutional series of malignant matrix-producing breast tumors Breast 2013 11 13 18 10.1016/j.breast.2012.09.010 23084962

